# Studying the System-Level Involvement of MicroRNAs in Parkinson's Disease

**DOI:** 10.1371/journal.pone.0093751

**Published:** 2014-04-01

**Authors:** Paulami Chatterjee, Malay Bhattacharyya, Sanghamitra Bandyopadhyay, Debjani Roy

**Affiliations:** 1 Department of Biophysics, Bose Institute, Acharya J.C. Bose Centenary Building, Kolkata, India; 2 Department of C.S.E., University of Kalyani, Kalyani, Nadia, India; 3 Machine Intelligence Unit, Indian Statistical Institute, Kolkata, India; University of Naples Federico II, Italy

## Abstract

**Background:**

Parkinson's Disease (PD) is a progressive neurologic disorder that affects movement and balance. Recent studies have revealed the importance of microRNA (miR) in PD. However, the detailed role of miR and its regulation by Transcription Factor (TF) remain unexplored. In this work for the first time we have studied TF-miR-mRNA regulatory network as well as miR co-expression network in PD.

**Result:**

We compared the 204 differentially expressed miRs from microarray data with 73 PD related miRs obtained from literature, Human MicroRNA Disease Database and found a significant overlap of 47 PD related miRs (*p*-value<0.05). Functional enrichment analyses of these 47 common (Group1) miRs and the remaining 157 (Group2) miRs revealed similar kinds of over-representative GO Biological Processes and KEGG pathways. This strengthens the possibility that some of the Group 2 miRs can have functional roles in PD progression, hitherto unidentified in any study. In order to explore the cross talk between TF, miR and target mRNA, regulatory networks were constructed. Study of these networks resulted in 14 Inter-Regulatory hub miRs whereas miR co-expression network revealed 18 co-expressed hub miRs. Of these 32 hub miRs, 23 miRs were previously unidentified with respect to their association with PD. Hierarchical clustering analysis further strengthens the roles of these novel miRs in different PD pathways. Furthermore hsa-miR-92a appeared as novel hub miR in both regulatory and co-expression network indicating its strong functional role in PD. High conservation patterns were observed for most of these 23 novel hub miRs across different species including human. Thus these 23 novel hub miRs can be considered as potential biomarkers for PD.

**Conclusion:**

Our study identified 23 novel miR markers which can open up new avenues for future studies and shed lights on potential therapeutic targets for PD.

## Introduction

Parkinson's Disease (PD) is the second most prevailing neurodegenerative disorder in the world after Alzheimer's disease (AD) [1]. The histological hallmark of PD is accumulation of fibrillar inclusions named Lewy bodies in the dopaminergic neurons. Lewy body accumulation leads to the malfunction and death of those dopamine producing nerve cells in the mid brain region, mainly in the substantia nigra (SN) [2]. Neurotransmitter dopamine transmits messages to the part of the brain that control movement and coordination. Thus loss of dopamine leads to motor system disorder, leaving a person unable to control movement normally [3]. Elderly persons throughout the globe are mostly affected by PD having symptoms like poor memory, tremor, bradykinesia, rigidity or stiffness of the limbs, impaired balance and postural instability [4]. It is one of the chronic and progressive movement disorders in which symptoms get worsen over time. Though long studied but still there is no cure for PD. The present treatments only reduce the extent of the symptoms but contribute very little to the halt of disease progressions [5].

One of the main reasons behind this inadequate quantitative treatment method is the lack of reliable diagnostic tools for PD. Present treatment of PD solely depends on clinical symptoms which appear in most of the cases at a very later stage when most of the (60–70%) dopaminergic neurons are already lost [6]. Here comes the need of incorporation of molecular markers in the diagnosis process which can aid proper detection at an earlier stage and slow down its progression.

MicroRNAs (miRs) are short noncoding RNA sequences (of ∼22 nt length) that act as post-transcriptional regulators of protein-coding genes by binding mainly to their 3′ untranslated region, leading to mRNA degradation or translational inhibition [7]. The mode of regulation of the target mRNAs depends on the sequence complementarity between the miR and the mRNA. Thus miRs play a key role in modulating diverse cellular processes. Transcriptomic analysis of different brain regions of PD patients revealed that miRs play an important role in PD progression and pathogenesis [8]. However the underlying molecular processes regarding the function of miRs in PD are still not clear. Altered expression of miRs has been implicated in other neurodegenerative diseases like AD and Huntington Disease (HD). miR biomarkers have been identified to play a crucial role in these diseases [9], [10]. It is now evident that miR- mediated regulation is an emerging field of therapeutic approaches [11].

**Figure 1:**
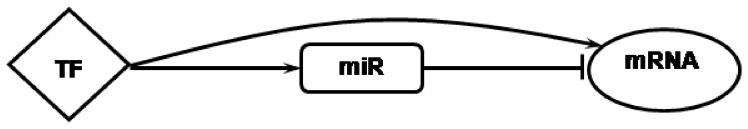
Regulatory relationship between TF, miR and mRNA. TFs and miRs often function in a coupled way. TFs can regulate the transcription of both the miR and its target mRNA by binding to their respective promoter region, while miRs regulate gene's post-transcription by binding to the 3′ untranslated region (UTR) of target mRNA.

Transcription Factors (TF) are proteins that can regulate the transcription of genes by binding to their upstream regulatory regions [12]. It has been proposed that not only protein-coding genes, but noncoding miR expressions are also tightly regulated by several TFs [13]. Since TFs and miRs are both part of a common regulatory network, they often function in a coupled manner. TFs can regulate the transcription of both the miR and its target mRNA by binding to their respective promoter region. miRs in turn regulate the post-transcriptional regulation of gene by binding to the 3′ untranslated region (3′ UTR) of target mRNA (**Figure 1**).

The simultaneous examination of such transcriptional/post-transcriptional regulatory networks comprising TFs, miRs and the target genes have emerged as a powerful tool to understand biological events and identify possible biomarkers [14]. A recent study of such miR-TF-mRNA regulatory network in Ovarian Cancer patients identified the transcriptome biomarkers associated with Ovarian Cancer survival and recurrence [15]. The miR-TF-gene regulatory networks have been studied for several types of human cancers such as colorectal and breast cancer [16], [17]. miR and TF mediated regulatory networks in Glioblastoma identified the main regulation format consisting of miRs, TFs and their target genes [18]. However, no such studies are available for PD.

In this work for the first time we have studied the TF-miR-mRNA regulatory network of PD. The primary aim of our work was to integrate transcriptomic and system biological approach to study the cross-talk of TF, miR and their targeted mRNAs in PD. In addition we have built the co-expression network and studied the co-expression pattern of PD related miRs. In our TF-miR-mRNA regulatory network we have placed the miR in the middle layer considering the role of miRs as intermediate regulatory hubs. Thus our regulatory network is different from the previously studied networks which have placed the TF in the middle layer of regulation. In this way our study identified 23 novel hub miRs which were not previously reported to be associated with PD. Moreover, hsa-miR-92a appeared as a common hub in both regulatory and co-expression network indicating its strong functional role in PD. These hub miRs can be considered as possible biomarkers for PD which can shed insight into possible therapeutic targets for PD.

## Results

### Grouping of the DE miRs

**Figure 2:**
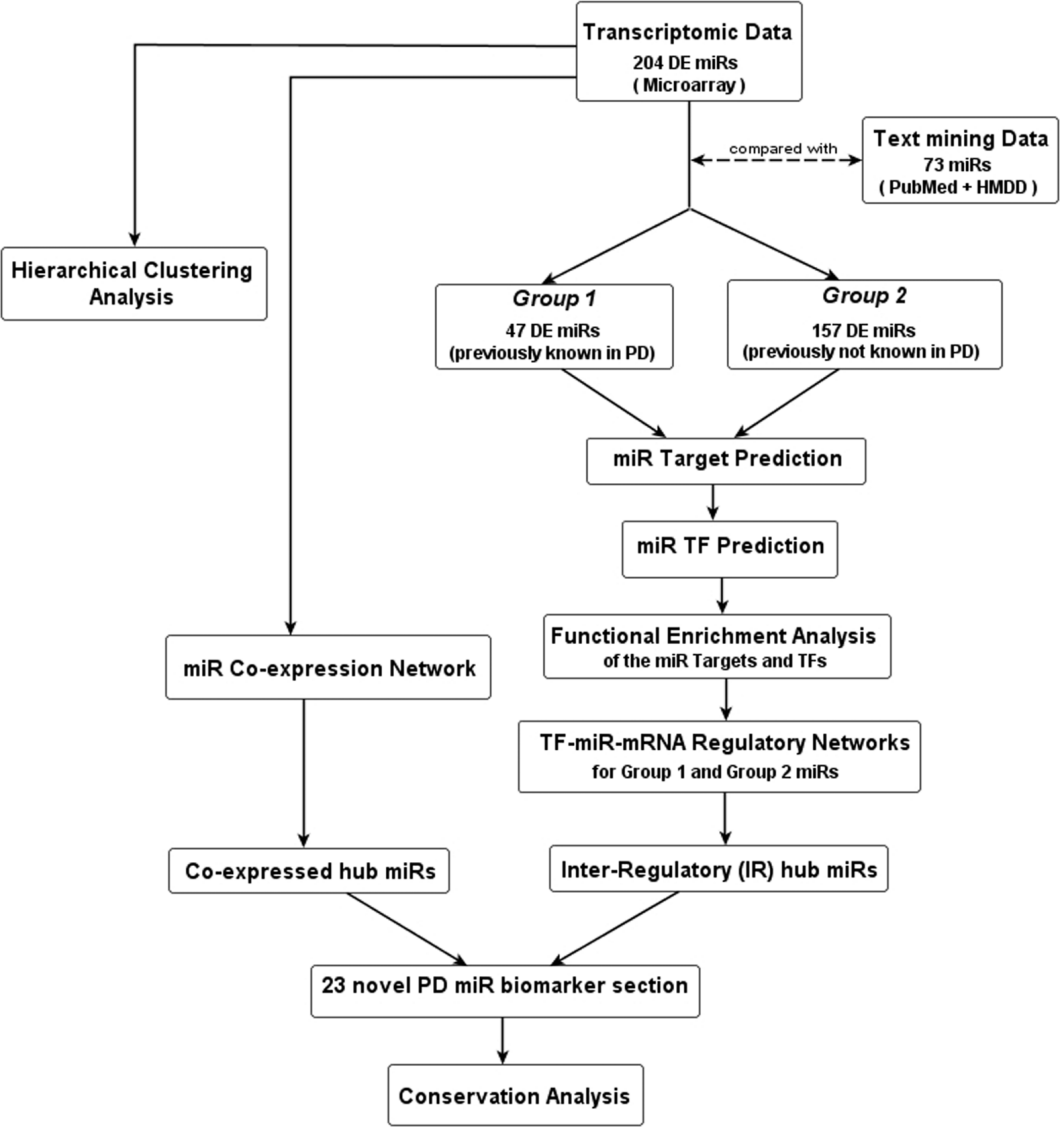
Flowchart depicting the workflow of this study.

**Figure 3:**
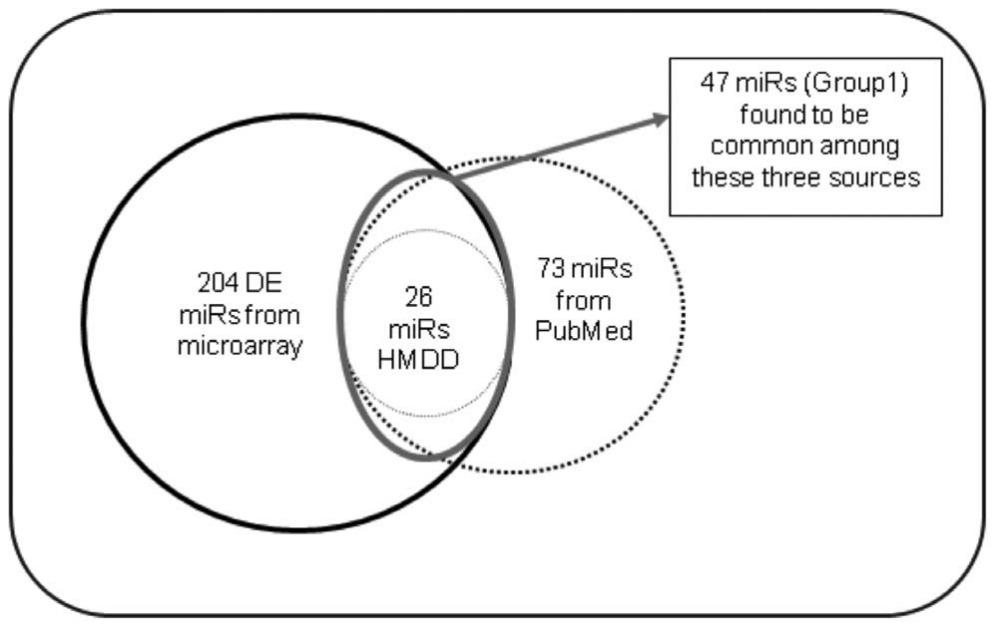
Collection of data from two different sources - miR microarray and text mining. We obtained 204 DE miRs from microarray expression data. Text mining incorporated information of 73 miRs which were reported to be linked with PD. This 73 miRs included 26 miRs from HMDD and 47 miRs from PubMed. Comparison of these transcriptomic and text mining data revealed a significant overlap of 47 PD related miRs, which were termed as Group1 and the remaining 157 miRs (out of the 204 miRs) were termed as Group 2 which were not previously reported to be associated with PD.

**Figure 2** highlights the workflow of our analysis. We identified the differentially expressed (DE) miRs between PD and control patients by applying the Significance Analysis of Microarray (SAM) with FDR value 0.3% [19]. We found 204 DE miRs. To validate our findings, we compared the 204 DE miRs with those 73 miRs found from text-mining (PubMed and HMDD) and found a significant overlap (*p*-value <0.05) of 47 PD related miRs (**Figure 3**). On the basis of this comparison, we divided the 204 DE miRs into two groups, **Group 1**, containing the common 47 miRs which were previously reported to be associated with PD in different literatures or databases and **Group 2**, containing the rest 157 miRs which were not previously found to be associated with PD but found to be DE in our study. This suggests that Group 2 can possibly contain novel miRs that are responsible for the etiology of PD but still unidentified in any study.

### Functional Annotation and Enrichment Analysis of the miR Targets

The mRNA targets of each of the two groups (Group1 and Group2) were determined from the TarmiR 1.0 platform (http://www.tarmir.rgcb.res.in/). We used the shared target list between three servers DIANA microT, miRanda and TargetScan and selected the target genes with DIANA miTG score equal or greater than 20 as the highly reliable targets. miR Target prediction of the two miR groups revealed that 1127 unique mRNAs were targeted by the 47 miRs present in Group1 whereas the number of unique mRNA targets for Group2 was 1227. We analyzed the functional property of these two target groups (1127 targets for Group1 and 1227 targets for Group2) using the DAVID Bioinformatic Resources (http://david.abcc.ncifcrf.gov/home.jsp) [20]. We selected SP_PIR_KEYWORDS functional annotation category because maximum number of our target genes (47.3%) were involved in this category. It was found that both the miR groups targeted similar functional mRNAs (**Table 1**). Most of the targets of these two groups belonged to the Phosphoprotein family indicating the possible role of these miRs in various signaling cascades. This indication was further strengthened by the results of enrichment analysis of the miR targets. It was found that these targets were indeed associated with various signaling pathways such as MAPK signaling pathway (hsa04010), mTOR signaling pathway (hsa04150), Adipocytokine signaling pathway (hsa04920), TGF-beta signaling pathway (hsa04350), Neurotrophin signaling pathway (hsa04722) etc (**Table 2**). Proteins involved in alternative splicing, transcriptional regulation and transcription process are also present in the top 5 functional target classes.

**Table 1:** Top 5 Functional Properties associated with the mRNA targets of Group1 and Group2 miRs obtained from DAVID Bioinformatic Resources (http://david.abcc.ncifcrf.gov/home.jsp) [20].

mRNA targets for Group1miRs				mRNA targets for Group2 miRs			
Term	Count	%	*p*-value	Term	Count	%	*p*-value
**Phosphoprotein**	644	5.675009	4.79E-43	**Phosphoprotein**	729	5.7738	1.36E-57
**Alternative splicing**	593	5.22559	1.01E-22	**Transcription regulation**	243	1.9246	1.03E-23
**Transcription regulation**	201	1.771237	1.84E-14	**Transcription**	244	1.93252	9.59E-23
**Transcription**	203	1.788861	4.38E-14	**Alternative splicing**	625	4.950103	1.80E-19
**Triple helix**	17	0.149806	2.09E-12	**Nucleus**	395	3.128465	2.57E-17

**Table 2:** Result of the Gene Ontology (GO) analysis for the miR TFs and target mRNAs of Group 1 and Group 2 miRs obtained from FatiGO (http://www.fatigo.org/) [21].

	No. of terms associated with Target genes		No. of terms associated with TFs	
	Group1	Group2	Group1	Group 2
**GO Biological Process**	595	1253	2075	2110
**GO Cellular Component**	79	134	217	217
**GO Molecular Function**	168	413	867	900
**KEGG PAthways**	40	88	188	192

In order to study the most significant GO terms (biological processes, molecular functions, cellular components) and KEGG pathways associated with these DE miRs, the target genes for Group1 and Group2 miRs were separately subjected to Functional enrichment analysis. FatiGO, a module in Babelomics 4.3.0 (http://www.fatigo.org/), was used to extract the most over-representative GO terms (Biological Process, Cellular Component and Molecular Function) for the groups of genes under observation with respect to the whole genome taken as the reference background set (*p*-value <0.05) (**Table 2**) [21].

Enrichment analysis of mRNA targets identified that Group1 and Group 2 miRs have similar biological processes and KEGG pathways. Cell development (GO:0048468), Neurogenesis (GO:0022008), Neuron differentiation (GO:0030182), Negative regulation of Gene expression (GO:0010629), Protein phosphorylation (GO:0006468) were among the highly significant biological processes shared by two groups (**Table S1**). While MAPK signaling pathway (hsa04010), mTOR signaling pathway (hsa04150), Endocytosis (hsa04144), Long-term potentiation (hsa04720), Dilated cardiomyopathy (DCM) (hsa05414), TGF-beta signaling pathway (hsa04350), Adipocytokine signaling pathway (hsa04920) etc were the highly significant KEGG pathways shared by the two groups (**Table 3**). This strengthens the possibility that Group 2 miRs can have possible functional role in PD progression. This established the significance of the entire set of DE miRs found by SAM and therefore this dataset is reliable for performing system-level analysis of PD. Interestingly **Table 3** also pointed out that there are several disease pathways associated with PD such as cancer pathways (hsa05200) and cardiovascular disease pathways. The association of PD and Cancer has been validated by several previous studies which showed that PD patients are at a higher risk for certain cancers (Melanoma, Prostate Cancer etc.) [22]. A very recent study reported an interesting link between Parkinson's disease and heart failure which can be used as a validation for our finding [23].

**Table 3:** Functional Enrichment analysis of the miR targets - top 20 most significant KEGG pathways associated with the target mRNAs of Group1 and Group2 miRs.

Group 1			Group 2		
ID	Name	*p*-value	ID	Name	*p*-value
**hsa04120**	Ubiquitin mediated proteolysis	4.02E-07	**hsa04010**	MAPK signaling pathway	1.58E-10
**hsa04010**	MAPK signaling pathway	5.01E-07	**hsa04720**	Long-term potentiation	1.82E-09
**hsa04150**	mTOR signaling pathway	7.42E-07	**hsa04012**	ErbB signaling pathway	2.14E-09
**hsa05200**	Pathways in cancer	8.97E-06	**hsa04114**	Oocyte meiosis	6.16E-09
**hsa04930**	Type II diabetes mellitus	9.28E-06	**hsa04350**	TGF-beta signaling pathway	2.01E-07
**hsa04144**	Endocytosis	1.28E-05	**hsa04912**	GnRH signaling pathway	2.23E-07
**hsa04350**	TGF-beta signaling pathway	2.63E-05	**hsa04914**	Progesterone-mediated oocyte maturation	2.51E-07
**hsa04722**	Neurotrophin signaling pathway	3.14E-05	**hsa04144**	Endocytosis	9.03E-07
**hsa05412**	Arrhythmogenic right ventricular cardiomyopathy (ARVC)	4.94E-05	**hsa05414**	Dilated cardiomyopathy (DCM)	1.57E-06
**hsa05414**	Dilated cardiomyopathy (DCM)	5.78E-05	**hsa04150**	mTOR signaling pathway	2.46E-06
**hsa05410**	Hypertrophic cardiomyopathy (HCM)	1.35E-04	**hsa04540**	Gap junction	6.83E-06
**hsa04720**	Long-term potentiation	1.40E-04	**hsa04916**	Melanogenesis	1.74E-05
**hsa04960**	Aldosterone-regulated sodium reabsorption	1.64E-04	**hsa04270**	Vascular smooth muscle contraction	2.03E-05
**hsa04920**	Adipocytokine signaling pathway	3.52E-04	**hsa04920**	Adipocytokine signaling pathway	4.10E-05
**hsa04115**	p53-signalling-pathway	3.87E-04	**hsa04020**	Calcium signaling pathway	6.46E-05
**hsa04520**	Focal adhesion	4.48E-04	**hsa05410**	Hypertrophic cardiomyopathy (HCM)	8.41E-04
**hsa05210**	Colorectal cancer (CRC)	5.66E-04	**hsa04062**	Chemokine signaling pathway	9.41E-04
**hsa05220**	Chronic myelogenous leukemia (CML)	9.22E-04	**hsa05120**	Epithelial cell signaling in Helicobacter pylori infection	6.24E-03
**hsa04142**	Lysosome	1.21E-03	**hsa01040**	Biosynthesis of unsaturated fatty acids	8.51E-03
**hsa04012**	ErbB signaling pathway	1.01E-02	**hsa00071**	Fatty acid metabolism	1.01E-02

### Transcription Factor Prediction and Enrichment Analysis of TFs

In order to study the transcriptional regulation on miR expression, TF information for all of the 204 DE miRs were collected from TransmiR Platform (http://202.38.126.151/hmdd/mirna/tf/) [24]. This database contains experimentally validated TF information for different species along with the possible role of particular TF on each miR expression. 41 TFs were obtained for Group 1 (47 miRs) and 56 TFs were obtained for Group 2 (157 miRs).

In order to explore the functional association of TFs in different KEGG pathways we performed FatiGo analysis. The results indicated that both Group 1 and Group 2 DE miRs were regulated by similar TFs contained in similar GO terms and KEGG pathways (**Table S2**). The possibility that Group 2 can contain novel miRs responsible for PD progression is further strengthened by the results of the ontology analysis as the Group2 miRs target similar kinds of mRNAs like Group1 miRs and they are regulated by similar kinds of TFs.

### Regulatory Network Construction and Inter Regulatory hub miR Selection

To identify the regulatory relationship between the TFs, miRs and mRNAs, a regulatory network was constructed for each Group1 and Group 2 miRs. miRs, associated with the highly significant top 20 biological processes, were selected for this network construction. Enrichment analysis in FatiGO revealed that among the 47 miRs in Group1, 29 were associated with top 20 biological process and in Group2, 59miRs out of 157 miR were associated with the top20 biological processes for Group2 (**Table S3**).

**Figure 4:**
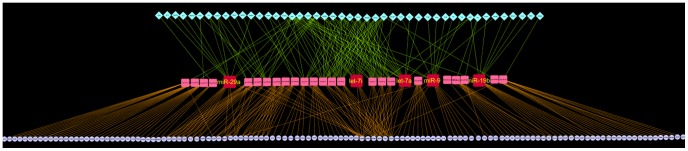
Tripartite regulatory network for Group 1 miRs. This network represents the molecular cross talk between TFs, miRs and mRNAs in PD. Square nodes in the middle layer represent miRs, diamond nodes in the upper layer representing validated TFs of respective miRs and circular nodes in the lower most layer represent mRNA targets of the miRs. Here regulation goes down from TFs to miRs and then miRs to mRNAs. TFs regulate the transcription of miRs whereas miRs regulate the translation process of target mRNAs. miRs with highest intermediate regulatory measure were denoted as IR hubs. The 5 IR hub miRs in the middle layer have been enlarged for proper visualization. 29 already known PD related miRs, associated with the top 20 most significant GO Biological Processes, were used to build this network. This network was constructed in Cytoscape interface [26].

**Figure 5:**
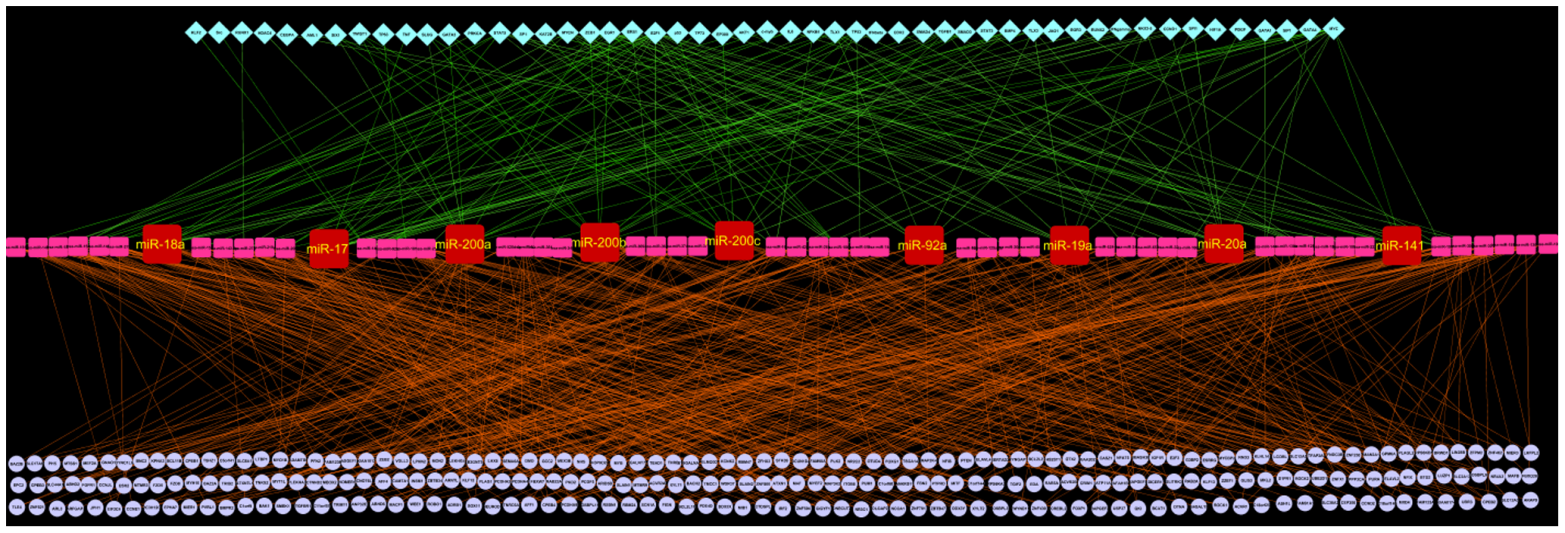
Tripartite regulatory network for Group 2 miRs. 59 Group 2 miRs associated with the top 20 most significant GO Biological Processes were used to build this network. This network represents the molecular cross talk between TFs, miRs and mRNAs in PD. Square nodes in the middle layer represent miRs, diamond nodes in the upper layer representing validated TFs of respective miRs and circular nodes in the lower most layer represent mRNA targets of the miRs. Here regulation goes down from TFs to miRs and then miRs to mRNAs. TFs regulate the transcription of miRs whereas miRs regulate the translation process of target mRNAs. miRs with highest intermediate regulatory measure were denoted as IR hubs. The 9 IR hub miRs in the middle layer have been enlarged for proper visualization. These IR hubs represent the novel hub miRs which are not reported previously to be linked in PD. This network was constructed in Cytoscape interface [26].

We observed the regulatory network as a tripartite network oriented in three layers from top to bottom in which TFs were present in the uppermost layer, miRs were in the middle layer and mRNAs were present in the lowermost layer (**Figure 4** **,** **Figure 5**). Here the regulation goes down from TF to miRs and then miRs to mRNAs. Thus the regulatory network describes the crosstalk among the TFs, miRs and their target mRNAs. On the basis of the number of TF (in-degree) and target mRNA (out-degree) per miR we identified the hub nodes, miRs that play the most important role in this tripartite regulatory network.

miRs present in the middle layer play a very important role in Intermediate Regulation. By channeling the vast amount of regulatory information (in terms of signals) from TFs to mRNAs they function as bottleneck points in the tripartite network. So identification of these intermediate regulatory (IR) points in the network can be considered as a novel measure for detecting hub miRs.

We used this IR measure to identify the potential IR hub miRs. In case of regulatory network for Group1 miRs we found that the highest IR value was 90 where the in-degree measure (*m*) was 9 and out-degree measure (*n*) was10 (**Table 4**). We selected the miRs having mXn value greater than equal to 70. The 5 IR hub miRs identified in this process were hsa-miR-29a, hsa-miR-9, hsa-let-7a, hsa-let-7i and hsa-miR-19b showing a high association with various signaling pathways.

**Table 4:** IR hub miRs identified on the basis of intermediate regulation measure from the regulatory network of Group1 miRs which are already reported to be associated with PD.

miRs	In-degree (m)	Out-degree (n)	Intermediate Regulation (mXn)
**hsa-miR-29a**	9	10	90
**hsa-miR-9**	8	10	80
**hsa-let-7a**	8	10	80
**hsa-let-7i**	7	10	70
**hsa-miR-19b**	7	10	70

In case of regulatory network for Group 2 miRs we found 9 hub miRs. The highest IR value in this case was 130 (where m is 13 and n is 10) which was higher than the highest IR value of Group1 (**Table S4**). We selected miRs with IR value greater than equal to 70 (**Table 5**). The 9 IR hub miRs in Group 2 regulatory network which play an important role in inter-regulatory signal transduction were hsa-miR-200c, hsa-miR-200b, hsa-miR-200a, hsa-miR-17, hsa-miR-19a, hsa-miR-20a, hsa-miR-18a, hsa-miR-141and hsa-miR-92a. Thus the tripartite regulatory network identified novel hub miRs which were not reported earlier in association with PD and hence can be considered as potential target for future study.

**Table 5:** IR hub miRs identified on the basis of intermediate regulation measure from the regulatory network of Group2 miRs which are not previously reported to be associated with PD.

miRs	In-degree (m)	Out-degree (n)	Intermediate Regulation (mXn)
**hsa-miR-200c**	13	10	130
**hsa-miR-200b**	12	10	120
**hsa-miR-200a**	12	10	120
**hsa-miR-17**	10	10	100
**hsa-miR-19a**	10	10	100
**hsa-miR-20a**	10	10	100
**hsa-miR-18a**	9	10	90
**hsa-miR-141**	7	10	70
**hsa-miR-92a**	7	10	70

In order to identify the TFs that were regulating maximum number of miRs, we visualized the TF-miR network corresponding to Group1 and Group 2 (**Figure S1, Figure S2**). Out-degree analysis of TFs in these networks revealed that in case of Group1 miRs- MYC, EIF2C2, LIN28B, LIN28, NFKB1 were among the TFs possessing high functional role in regulating PD miRs. In case of Group 2 miRs- MYC, MYCN, ERS1, E2F1, NKX2-5, SPl1, TGFB1, TLX3, EGR1, STAT5 were among the highly regulatory TFs.

### Hierarchical Clustering Analysis

Next we followed Hierarchical clustering (Hclust) method to arrange the 204 DE miRs into groups based on their similarity in expression profile to gain some meaningful biological insight. Hclust Analysis revealed 6 clusters - cluster 1 containing 3 miRs, cluster 2 containg 62 miRs, cluster 3 containing 99 miRs, cluster 4 containg 25 miRs, cluster 5 containg 1 miR and cluster 6 containing 14 miRs (**Table S5**). The mRNA targets of each of the six clusters were determined as described previously from the TarmiR 1.0 platform (http://www.tarmir.rgcb.res.in/) using a specific threshold value.

The set of target list for each cluster was then separately uploaded to FatiGo to identify the significant GO terms and KEGG pathways associated with each cluster. Our aim was to find out the unique biological term associated with each cluster which we can point out as the characteristic property of that cluster.

Enrichment analysis for the six clusters revealed that most of the over representative pathways were shared between different clusters and very few pathways were uniquely associated with a single cluster. Cluster 1 and 5 were not associated with any significant GO terms and KEGG pathway. So we continued our analysis with the remaining 4 clusters. Pathway analysis of Cluster 2, 3, 4 and 6 revealed that Axon Guidance (hsa04360), Ubiquitin mediated proteolysis (hsa04120), Pathways in cancers (hsa05200), Regulation of actin cytoskeleton (hsa04810) etc were highly prevalent among four clusters (**Table S6**). Besides Focal Adhesion (hsa04510), MAPK signalling pathway (hsa04010), Glioma (hsa05214), Neurotrophin signalling pathway (hsa04722) were highly significant in most of the clusters. Previous studies have identified the association of these pathways with PD [25]. But our study was first to find out the roles of these new miRs, present in the Group 2, in these PD pathways. By this, Hclust analysis further strengthens the significance of these 204 DE miRs and emphasizes the association of these unreported miRs with PD.

### Co-expression Network Analysis

**Figure 6:**
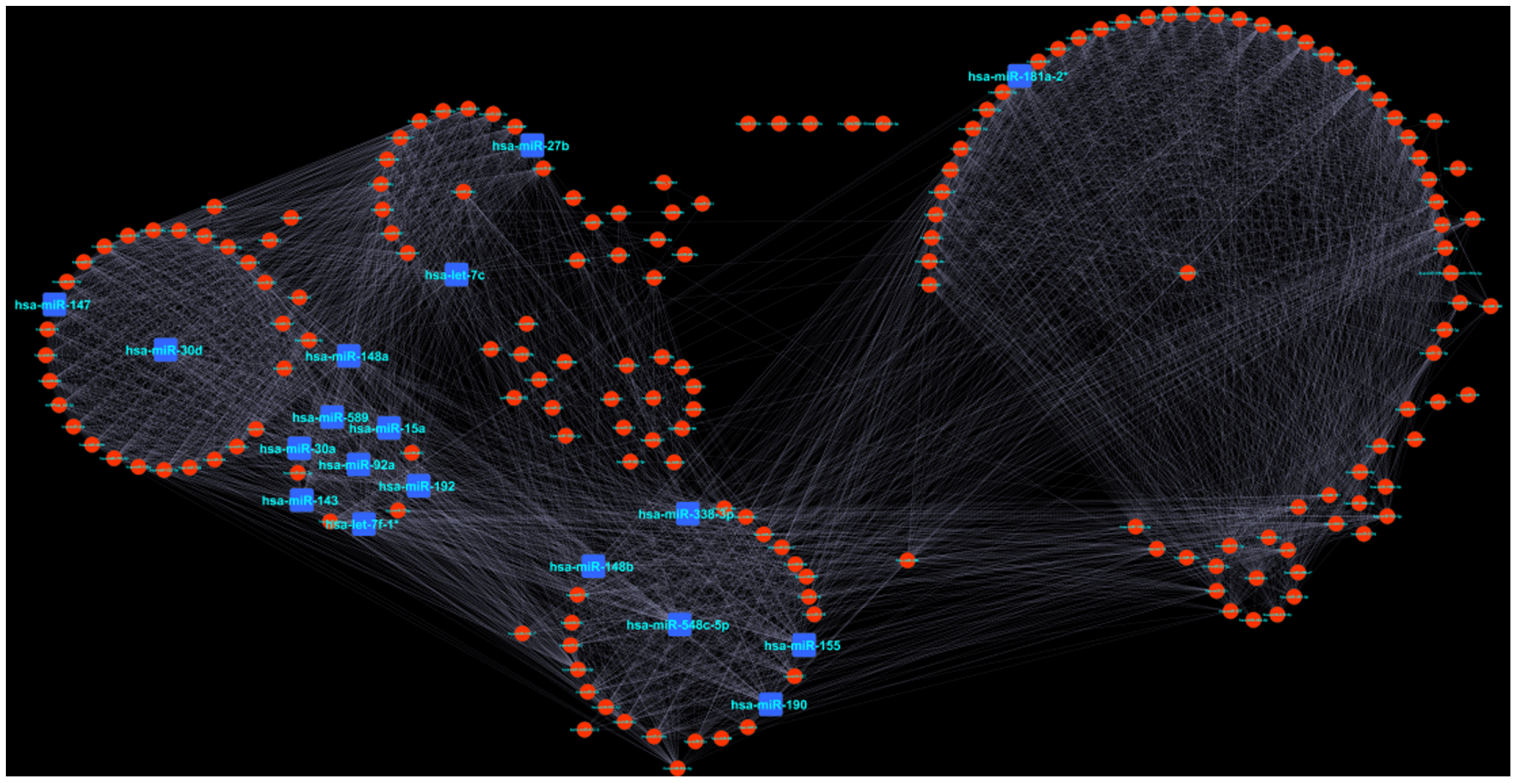
miR-miR co-expression network. This network was built with the 195 DE miRs which have pearson correlation co-efficient greater than 0.9. Here nodes correspond to miRs, and edges between miRs represent significant co-expression relationships. Top 18 co-expressed hub miRs were represented as square nodes, of which 4 hub miRs belonged to Group 1. The remaining 14 co-expressed hub miRs were novel miRs which were not previously reported to be associated with PD. This network was constructed in Cytoscape interface [26].

For co-expression network analysis with the 204 DE miRs, we first obtained the pairs of miRs that have *r* value greater than 0.9 and this yielded 3730 miR pairs. Out of the 204 DE miRs, we found 195 miRs were involved in these 3730 pairs. We visualized the entire network between them (**Figure 6**) using the open source network visualization software Cytoscape version 2.8.3 [26]. We analyzed four topological properties (degree, betweenness, eccentricity and clustering coefficient) of these 195 nodes (miRs) present in the co-expression network using the tYNA (http://tyna.gersteinlab.org/) web interface [27].

**Degree** or **connectivity** is an important topological parameter of a network which represents the number of connections or edges of a particular node [28].

**Betweenness Centrality** (BC) is another topological parameter of a node. It is given by the expression: 
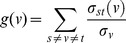



Where 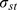 represents the total number of shortest paths from node *s* to node *t*, and 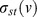 is the total number of shortest paths that pass through. BC quantifies the flow of information through a node in the network. It specifies how a node influences the communication among other nodes. Therefore with the increase of BC value, the importance of a node in a network increases [29].

The **eccentricity** of a node is the length of its maximum shortest paths. The maximum non-infinite length of a shortest path between *n* and another node in the network is denoted as its eccentricity. If *n* is an isolated node, the value of this attribute is zero. Sometimes maximum node eccentricity is used to define the network diameter. It can be thought of as how far a node is from the node most distant from it in the graph [30].

In undirected networks, the **clustering coefficient** (*C_n_*) of a node *n* is defined as 
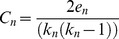



Where *k_n_* is the number of neighbors of *n* and *e_n_* is the number of connected pairs between all neighbors of *n* [31]. In both cases, the clustering coefficient is a ratio *N*/*M*, where *N* is the number of edges between the neighbors of *n*, and *M* is the maximum number of edges that could possibly exist between the neighbors of *n*. The clustering coefficient of a node is always a number between 0 and 1.

The global node statistics for overall Co-expression Network were obtained from tYNA (**Table 6**). The 195 miRs exhibited a varied degree distribution with highest degree of 79 and lowest degree of 1. The Average number of degrees present is 38.2 with median 45 and standard deviation 22.064. We considered the top 18 nodes (according to the maximum degree value) as the hub or essential nodes of this network because these 18 nodes possessed more than 80% of the total connectivity (degree >63.2).

**Table 6:** Node Statistics obtained from the tYNA (http://tyna.gersteinlab.org/) web interface for the overall co-expression network built with the 195 highly correlated miRs [27].

Degrees				Clustering Coefficient				Eccentricities				Betweenness			
Avg	SD	Min	Max	Avg	SD	Min	Max	Avg	SD	Min	Max	Avg	SD	Min	Max
38.26	22.01	1	79	0.70	0.22	0.00	1.00	5.37	0.98	2	8	148.19	228.77	0.00	1,596.46

Among the 18 hub miRs found in the co-expression network analysis, 4 miRs (hsa-miR-30a, hsa-let-7c, hsa-let-7f-1 and hsa-miR-147) were previously linked to PD. The remaining 14 miRs belonged to Group2 miRs, in other word their association were unreported in PD. The topological parameters of these 14 nodes (**Table 7**) showed that the highly connected nodes (hsa-miR-190, hsa-miR-155, hsa-miR-338-3p) also have very high BC value and low eccentricity value. This indicated their critical position and functional importance in the network in terms of information flow and hence their importance in PD progression.

**Table 7:** Topological Properties of the 18 hub miRs identified in the co-expression Network.

Hub nodes	Degree	Betweenness	Eccentricities	Clustering Co-efficient
**hsa-miR-190**	79	873.58844	4	0.548847777
**hsa-miR-155**	77	700.1562131	4	0.568694463
**hsa-miR-148a**	74	183.9454213	5	0.656053314
**hsa-miR-92a**	74	184.0570282	5	0.65901518
**hsa-miR-338-3p**	73	692.74642	4	0.5304414
**hsa-miR-143**	71	160.4184355	5	0.671227364
**hsa-miR-30a** #	70	155.4571725	5	0.699792961
**hsa-miR-181a-2**	69	544.8636743	5	0.549019608
**hsa-miR-30d**	69	140.8784072	5	0.700341006
**hsa-miR-589**	69	159.8119127	5	0.709292413
**hsa-let-7c** #	67	736.5219806	5	0.674807779
**hsa-miR-148b**	67	578.5944648	4	0.54816825
**hsa-let-7f-1** #	66	132.8030013	5	0.71048951
**hsa-miR-15a**	66	227.562842	5	0.718414918
**hsa-miR-147**	65	434.2587801	5	0.701923077
**hsa-miR-192**	65	105.3789872	5	0.740384615
**hsa-miR-27b**	64	60.13700936	5	0.766865079
**hsa-miR-548c-5p**	64	53.89115547	5	0.771825397

# miR previously reported to be associated with PD.

To find out the biological significance of these co-expressed hub nodes we analyzed the targets of these 18 hub miRs (751 unique genes) obtained from the co-expression network using FatiGo. KEGG pathway analysis for the 18 hub miRs revealed somewhat similar results like Hclust analysis. The 18 hub miRs were shown to have significant association in several PD related pathways which further strengthened our findings (**Table 8**).

**Table 8:** Top 10 most significant KEGG pathways associated with the 18 hub miRs obtained from co-expression network analysis.

KEGG pathways	Name	*p*-value
**hsa04360**	Axon guidance	6.71E-10
**hsa04120**	Ubiquitin mediated proteolysis	1.44E-06
**hsa04114**	Oocyte meiosis	2.02E-06
**hsa04510**	Focal adhesion	2.62E-06
**hsa04350**	TGF-beta signaling pathway	6.08E-06
**hsa05200**	Pathways in cancer	7.16E-06
**hsa04010**	MAPK signaling pathway	2.10E-05
**hsa05222**	Small cell lung cancer	3.52E-05
**hsa05414**	Dilated cardiomyopathy (DCM)	5.45E-05
**hsa04912**	GnRH signaling pathway	9.11E-05

### Conservation Analysis

Our study identified 23 previously unreported disease markers for PD, 9 from TF-miR-mRNA regulatory network and 14 from miR co-expression network. Of these 23 miRs, hsa-miR-92a appeared as hub in both regulatory and co-expression network indicating its strong functional role in PD.

To investigate the importance of the newly identified hub miRs from an evolutionary perspective, we studied the conservation patterns of these 23 novel hub miRs. The PhastCons datasets of UCSC Genome Browser (http://genome.ucsc.edu/) was used for this purpose [32]. Here the evolutionary conservation of miR is measured through multiple sequence alignments of 46 vertebrate species. Through these alignments PhastCons Score is generated which ranges from 0–1000 [33]. PhastCons score assigned to 9 IR hub miRs showed very high evolutionary conservation of these hub miRs (**Table 9**). The high evolutionary conservation pattern of these IR hubs indicates that these may act as key regulators among conserved species. **Table S7** shows the PhastCons scores assigned to 15 co-expressed hub miRs. It was found that 11 out of the 14 co-expressed hub miRs were assigned medium to high PhastCons scores indicating their strong conservation among different species.

**Table 9:** Summary statistics for the conservation analysis obtained from the PhastCons dataset of UCSC Genome Browser (http://genome.ucsc.edu/) for the 9 novel IR hub miRs (which are not previously linked with PD) [32].

miR name	Position (obtained from miRBase) [35]	PhastCons Score		
		Smallest	Biggest	Average
**hsa-miR-200c**	chr12: 7072862–7072929	562	562	562
**hsa-miR-200b**	chr1: 1102484–1102578	314	566	440
**hsa-miR-200a**	chr1: 1103243–1103332	509	510	510
**hsa-miR-17**	chr13: 92002859–92002942	659	659	659
**hsa-miR-19a**	chr13: 92003145–92003226	720	720	720
**hsa-miR-20a**	chr13: 92003319–92003389	750	750	750
**hsa-miR-18a**	chr13: 92003005–92003075	720	720	720
**hsa-miR-141**	chr12: 7073260–7073354	558	558	558
**hsa-miR-92a** *	chr13: 92003568–92003645	750	750	750

* hsa-miR -92a appeared as a common hub in both regulatory and co-expression network.

**Figure 7:**
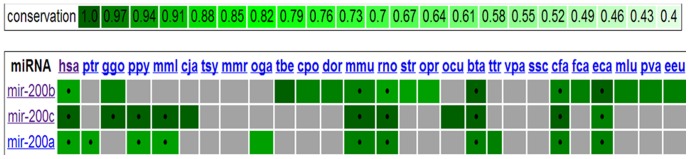
Conservational view of miR 200a, miR 200b and miR 200c in different species. This figure indicates high conservation pattern of these three novel IR hubs in different species including human. This information was obtained from miRNAviewer which presents a global view of homologous miR genes in many species [34]. The colored legend indicates the conservation level of each grouped miR. Grey box indicates that the miR was not identified in this genome, under stringent parameters. Symbols • indicate miRs registered in miRbase [35].

In order to acquire more evolutionary insight into the global conservational view of the known homologous miR genes in multiple species, a recently published web server microRNAviewer was used [34]. This includes the homologous miRs, related to each other by descent from a common ancestral DNA sequence, that are either included in miRbase v.16 [35] or were identified by a full cross-search using miRNAminer [36]. For a total of 49 species, the conservation scores are available for more than 2,300 miRs within a range of 0 to 1. The 9 novel IR hubs and the 14 co-expressed miR hubs were both analyzed using this tool. We obtained very high conservation scores for almost all of these 23 miRs, especially they are highly conserved in human. The conservational view of the novel IR hubs (miR-200a, miR-200b, miR-200c) in different organisms and the associated scores (pictorially) are highlighted in **Figure 7**. Besides, the screenshot of multiple sequence alignments for miR-200c is shown in **Figure S3** which displays a strong conservation pattern across different species. Therefore, our claims become stronger also from the evolutionary perspective.

**Figure 8:**
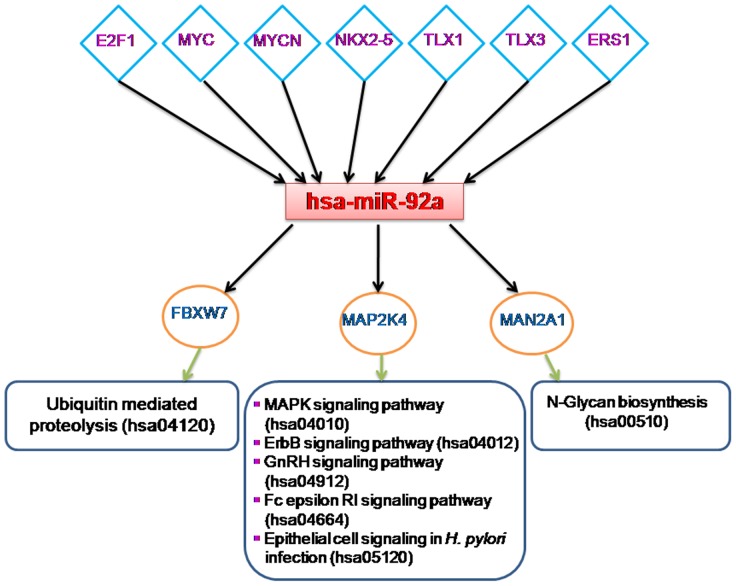
Association of TFs, target mRNAs and significant KEGG pathways with hsa-miR-92a. This miR appeared as a common hub between regulatory and co-expression network. Functional enrichment analysis strengthens its role in several PD related pathways.

**Figure 9:**
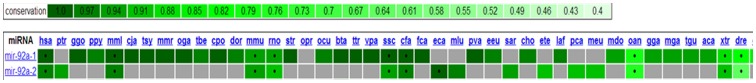
Conservational view of miR 92a in different species. According to miRbase information, miR 92a is presently denoted as miR 92a-2. This figure indicates high conservation pattern of this miR which was found to be a common hub between regulatory and co-expression network. The information regarding the conservation analysis was obtained from miRNAviewer which presents a global view of homologous miR genes in many species [34]. The colored legend indicates the conservation level of each grouped miR. Grey box indicates that the miR was not identified in this genome, under stringent parameters. Symbols • indicate miRs registered in miRbase [35].

In this way our study identified hsa-miR-92a as the common hub between regulatory and co-expression network suggesting its strong functional role in PD. Enrichment analysis of the mRNA targets further emphasized its association in several PD pathways (**Figure 8**). Moreover we found high conservation scores for this miR in different species (**Figure 9**). Therefore hsa-miR-92a can be considered as a possible biomarker for PD which is still unidentified in any study.

## Discussion

PD is one of the leading causes for progressive motor neuron disease in elderly persons and the prevalence of this clinical disorder is constantly growing worldwide. There is agreement about the fact that clinical distinction of PD is often challenging at an early stage which leads to the importance of identification of disease biomarkers for PD. In this study we have carried out a new kind of system-level analysis, to explore the involvement of microRNAs in the Parkinson's disease, which is fundamentally different than the existing studies.

Here, we have been able to combine transcriptomic and text mining approach to identify miR biomarkers in PD. In our work TF-miR-mRNA regulatory networks and miR-miR co-expression network were simultaneously analyzed and inter-regulatory measures were used for the first time to identify bottleneck IR hub miRs. In this way we identified 23 miRs previously not known to be associated with PD. Of these 23 miRs 9 were identified as IR hub miRs while the remaining 14 were identified as co-expressed hub miRs. The tripartite regulatory network comprising TF-miR-mRNA explored the crosstalk among the three molecular markers and identified the hub miRs which play an important role in inter-regulatory signal transduction. On the basis of intermediate regulation the regulatory network identified some novel hub miRs(hsa-miR-200c, hsa-miR-200b, hsa-miR-200a, hsa-miR-17, hsa-miR-19a, hsa-miR-20a, hsa-miR-18a, hsa-miR-141 and hsa-miR-92a) which were not reported earlier in association with PD and hence can be considered as potential target for future study.

Several previous studies have related miR-200 family in cancer metastasis and cancer progression [37]. They identified the role of miR-200 family in Epithelial to Mesenchymal transition (EMT) which is a crucial event in cancer metastasis. Studies have shown that it can regulate olfactory neurogenesis [38]. A recent study has also pointed out the role of miR-200 family in neural induction [39] which is the earliest step in neuronal development and a link of miR-200 has been established in neurodegeneration (in case of Drosophila) [40]. These findings deserve follow up exploration in human studies, so that the involvement of miR-200 family in PD can bring out some interesting features which can be helpful in PD therapeutics.

Surprisingly most of the 23 novel miRs were found to be associated with several cancer pathways such as Pancreatic Cancer (hsa-miR-200c [41], hsa-miR-141 [42]), Lung Cancer (hsa-miR-200c [43], hsa-miR-143 [44]), Colorectal Cancer (hsa-miR-200c [45], hsa-miR-338-3p [46]), Bladder Cancer (hsa-miR-200b, hsa-miR-200a, hsa-miR-141, hsa-miR-17, hsa-miR-27b) [47]-[49], Breast Cancer (hsa-miR-200b [50], hsa-miR-147 [51]), Esophageal Cancer (hsa-miR-200a, hsa-miR-141, hsa-miR-143, hsa-miR-15a) [52], [53], Prostate Cancer (hsa-miR-19a) [54], Oral Carcinoma (hsa-148b) [55], Cervical Cancer (hsa-miR-15a) [56], Gastric Cancer (hsa-miR-192) [57] etc. Previous studies have indicated that miR-200 family is highly expressed in Endometrial Carcinoma compared with that of normal endometrial tissues and could play an important role in cancer growth [58]. It has been found that hsa-miR-148a can be a potential therapeutic target for cancer therapy as this miR inhibits tumor growth [59]. Moreover, hsa-miR-181a-2 has been found to be up regulated in the head and neck cancer patients which may be considered as possible risk factor in these diseases [60]. Besides, hsa-miR-30d has been implicated in medulloblastoma pathogenesis [61]. As we have already mentioned that the association of PD and Cancer has been established by several previous studies [62]. Therefore our finding of these 23 miRs indicated that these miRs can be possible regulators in both of these diseases.

In addition to these cancer pathways, the 23 hub miRs were found to be associated with several other diseases. hsa-miR-200b has been found to be associated with the pathophysiology of autism [63]. Previous studies have established a link between hsa-miR-190 and the aggressive phenotype of neuroblastoma [64]. Moreover hsa-miR-19a, hsa-miR-20a, hsa-miR-17 and hsa-miR-155 have been reported to have a role in periodontal inflammatory pathways [65].

In our study, results of the functional enrichment analysis pointed out a close association between several cardiovascular disease pathways and PD. This was also validated by the finding that several of the hub miRs were previously related to heart diseases. Higher expression level of hsa-miR-143 has been found in pulmonary arterial hypertension [66]. Besides, a previous RNA sequencing study has found that hsa-miR-143 is differentially expressed in the right and left atria which may yield insight into the increased arrhythmogenesis of the left atria [67]. Furthermore, hsa-miR-192 has been identified as a predictive indicator of heart failure after acute myocardial infarction [68].

It is noteworthy to mention that hsa-miR-92a was found to be the hub miR in both regulatory and co-expression networks indicating its strong functional role in PD. hsa-miR-92a has been implicated as biomarker in several types of cancers (Pancreatic, Prostate, Ovarian Cancer etc) [69], [70]. Besides it has also been reported to be differentially expressed in the whole blood sample of patients with coronary artery disease which confirms hsa-miR-92a as a possible therapeutic target for cardiovascular diseases [71]. In this way with the help of a new network based approach our study proposed the exploration of several novel miR biomarkers for PD and shed light on the association of several disease pathways with PD.

This study was done with microarray data derived from blood samples of PD patients. We have not been able to incorporate brain specific PD miR expression data. However we have done extensive text mining and identified the overlap between microarray and text mining data. In this way we have been able to integrate both blood and brain specific PD related miRs to our initial dataset. Next generation sequencing data or ENCODE data can also be used in this kind of study. Besides, our study is heavily dependent on completeness and reliability of different databases. Any deviation from such criteria of databases will have an effect on our analysis.

## Conclusions

We performed a system-level analysis by studying the involvement of miRs in PD. In this study we have been able to combine transcriptomic and text mining approach to identify miR biomarkers in PD. In our work TF-miR-mRNA regulatory networks and miR-miR co-expression network were simultaneously analyzed and inter-regulatory measures were used for the first time to identify bottleneck IR hub miRs. In this way we identified 23 miRs previously not known to be associated with PD. Of these 23 miRs 9 were identified as IR hub miRs while the remaining 14 were identified as co-expressed hub miRs. It is noteworthy to mention that hsa-miR-92a was found to be the hub miR in both regulatory and co-expression networks indicating its strong functional role in PD. Furthermore, functional enrichment analysis of the mRNA targets associated with the 23 hub miRs including hsa-miR-92a strengthens their association with several PD related pathways. Moreover, our study shed light on several shared pathways associated with PD such as cardiovascular, cancer and different signaling pathways which strongly suggests that PD is a multifaceted disease that involves several molecular processes working in concert. Our study also identified very high conservation patterns for most of the 23 novel hub miRs across the species including human. Thus these 23 novel hub miRs can be considered as potential disease markers and therapeutic targets for PD. To our knowledge this is the first attempt on miR PD network biology which demonstrates how system level analysis provide insight into the intricate molecular cross talks associated with complex disease.

## Methods

### Data Collection

Microarray expression data was collected from Gene Expression Omnibus (GEO). Moreover, extensive text mining information about PD-associated miRs was collected from two sources - literature stored in PubMed (http://www.ncbi.nlm.nih.gov/pubmed) and Human MicroRNA Disease Database (HMDD) [72].

#### Microarray Data Collection from GEO

Exiqon miR microarray data of GSE16658 family was downloaded from GEO dataset browser (http://www.ncbi.nlm.nih.gov/geo/) [73]. Unlike other studies, this experiment was done on peripheral blood mononuclear cells (PBMCs) tissue samples of PD patients. Though PD primarily affects neurons in the mid brain region, information gathered from the study of PD blood samples can be proven useful to understand the pathobiology of PD because studies have indicated that the miR expression pattern in normal brain appears to be more similar to PBMCs than to other tissues [74]. The microarray data contained miR expression profiles obtained from PBMCs tissue of 19 PD patients and 13 controls (**Figure S4**). The clinical characteristics of the patients were given in the supplementary file (**Table S8**). Samples were labeled with Hy3 and Hy5 dyes. Hy3 was used for individual sample labeling and Hy5 was used for Common Reference Pool. The ExiMiR package was used for normalization of miR expression data [75]. Logarithmic conversion [log2 (Hy3/Hy5)] was performed in order to obtain the unified expression profile of the whole dataset which was used for further study.

#### Collecting Information about PD-associated miRs through text-mining

We browsed HMDD and collected the names of validated miRs that are listed as responsible for PD progression. We found 26 such miRs from HMDD. Further, we searched PubMed and collected reports about 47 more miRs that were already known to be associated with PD. For our search we used terms such as ‘microRNAs-PD’, ‘microRNA and Parkinson's Disease’, ‘microRNAs in Parkinson's disease’, etc. and the timeline was set with 2000 to 2013. In this way, we obtained a list of 73 miRs that have association with PD (**Table S9**).

### Differentially Expressed miR Selection

Significance Analysis of Microarray (SAM) was used to identify the differentially expressed (DE) miRs in the disease state which were either up-regulated or down-regulated. SAM calculates the False Discovery Rate (FDR) based on permutation analysis and relative difference of expression data. The test statistic is given by: 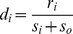



Where *d_i_* is the relative difference in gene expression, *r* is the linear regression coefficient of gene *i*, *s_i_* is the standard error of *r* and *s_o_* is a constant chosen to minimize the coefficient of variation of *d_i_*.

FDR is a statistical method that minimizes the number of incorrectly rejected null hypotheses in a test or it minimizes the number of false discoveries in a test. The lower the FDR the higher is the chance of finding a significant result with less number of false discoveries. Thus SAM assigns a score to each gene on the basis of change in gene expression relative to the standard deviation of repeated measurements.

In our study we found that at FDR value 0.3% (after sensitivity analysis) 204 miRs were DE among control and disease conditions. All of these 204 miRs were upregulated in PD. These 204 miRs were used to carry out the next phase of our study.

### Target Prediction

miR target prediction platform TarMiR 1.0 (http://www.tarmir.rgcb.res.in/) was used to identify the targets of the DE miRs. TarMiR1.0 is an integrated database that holds pre-computed microRNA target lists from nine commonly used miR target prediction servers along with target lists from the only server that provides a list of experimentally validated targets. Among them we used three servers DIANA micro T, miRanda and TargetScan to retrieve information from the pre-computed miR target lists in a customizable and comprehensive manner. These 3 prediction tools were selected because of the highest ratio of correctly predicted targets over other prediction tools. The shared/common targets of these three databases were selected for each miR. Gene list was then screened with the DIANA miR targeted gene (miTG) score. miTG score reflects the weighted sum of the scores of all conserved and nonconserved miR recognition elements on the 3′ UTR of the target mRNA. We selected the target genes with miTG score equal or greater than 20 as the highly reliable targets. Previous study by Satoh et Al. (2011) reported that targets with the miTG score (<20) indicates significantly lower precision score than the targets with miTG score more than 20, where precision score is an indicator of the correctness in predicted interactions [76]. Target prediction revealed that 1127 unique mRNAs were targeted by the 47 miRs present in Group1 whereas the number of unique mRNAs targets for Group2 was 1227. The mRNA targets of the 23 novel PD miR biomarkers were further validated from the TarBase 6.0 [77].

### Regulatory network Construction

In order to identify the regulatory relationship between the TFs, miRs and mRNAs, a regulatory network was constructed for each Group1 and Group 2 miRs. Both the networks were filtered based on the overrepresented GO biological processes. miRs, associated with the highly significant top 20 GO biological processes, were selected for this network construction. The TFs obtained from TransmiR database were used in this regulatory network. Top 10 target interactions (target genes with miTG score equal or greater than 20 were considered as the highly reliable targets) for each miR was shown in this network.

On the basis of the number of TF (in-degree) and target mRNA (out-degree) per miR we identified the hub nodes, miRs that play the most important role in this tripartite regulatory network. Let us assume the number of TFs per miR is m and number of target mRNA per miR is n (as we have chosen only top ten targets above the Diana miTG score 20, the upper limit of n is always 10). So each miR will be regulated by m number of TFs (signals) which will further dictate the miR to repress n number of targets. In other word each miR will exert its action via n number of possible ways. Thus the amount of regulatory information passing through a miR is mXn. We identified this in-degree, out-degree measure as one of the most important properties of hub miRs. This represents the intermediate regulatory (IR) property of a miR. It quantifies the flow of information through a node in the network. In our study nodes with such high IR values are considered as the IR hub nodes. These nodes are positioned in the central position in the tri partite regulatory network depicting the high amount of information flowing through them. We identified these IR hub nodes on the basis of the number of regulatory TFs and number of regulated mRNA connected to a particular miR. Higher this value higher is the probability of that miR to be involved in various signaling pathway hence can be probable candidate biomarker in a certain disease condition.

### Hierarchical Clustering (Hclust) analysis of the DE miRs

We wanted to classify the DE miRs into groups of miRs with similar expression patterns. Clustering is one of such methods that allow us to arrange data into groups of genes based on their similarity in expression profile to gain some meaningful biological inference about that set of genes. Clustering method can be hierarchical or non hierarchical. We approached the Hclust method that groups objects into clusters and specify relationships among objects in a cluster. It builds hierarchy of clusters like a phylogenetic tree. Hclust works on the idea that objects that are close to each other are more connected than to objects that are present at a distance. So in this method nearby objects are joined to form a cluster based on their distance [78].

Hclust can be of two types Agglomerative (starts with a single object and aggregates nearby objects into clusters) and Divisive (starts with the entire data set and divides it into small clusters). For a hierarchical agglomerative clustering procedure, each object is considered as a cluster. The first step is the calculation of distance between objects in a data matrix. We used the sample correlation method for calculating pair wise distance between two objects.

Given an m-by-n data matrix *X*, where row vectors are *x*_1_, *x*_2_, …, *x_m_*, the various correlation distances between the vector *x_s_* and *x_t_* are defined as follows

Correlation distance 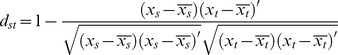



Where 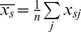 and 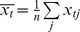.

Based on the pairwise distances between them, objects that are similar to each other are grouped into clusters. After this is done, pairwise distances between the clusters are re-calculated, and clusters that are similar are grouped together in an iterative manner until all the objects are included into a single cluster [79]. This information can be represented as a dendrogram, where the distance from the branch point is indicative of the distance between the two clusters or objects.

Since a cluster is composed of several objects there are several candidates to calculate the distance between two clusters. For this one needs to choose the linkage criterion for Hclust analysis. There are several methods of Hclust depending on the linkage criterion chosen. Popularly used methods are Single linkage clustering, complete linkage clustering, average linkage clustering or centroid linkage clustering. We used the average linkage clustering. Instead of calculating the minimum or maximum distance between two clusters, this method calculates the average distance between all possible pairs of objects in the two clusters.

The 204 DE miRs were subjected to Hclust (Agglomerative with average linkage clustering) analysis which revealed 6 clusters. These clusters were then subjected to GO analysis.

### Co-expression Network Analysis

Genes present in a same pathway often exhibit similar expression profiling under varied conditions. Therefore a group of genes that have similar expression pattern in different physiological conditions can be possible candidate of a similar pathway. These groups of genes represent a functional module and it is believed that each such module undergoes similar transcriptional regulation [80]. This is also relevant with miRs. If a group of miRs is found to have similar expression pattern over different conditions that can indicate their presence in a same functional module. In order to find such functional modules we studied the DE miR over control and disease conditions in the form of a co-expression network which is an undirected graph, where the graph nodes correspond to miRs, and edges between miRs represent significant co-expression relationships [81].

To create a co-expression network first of all we measured the Pearson correlation coefficient (*r*) for all possible combination of pairs of DE miRs over control and disease conditions. The basic formula for computing *r* is 
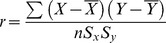



where *X* and *Y* are the scores of the variables whose Correlation Coefficient are being measured (here *X* and *Y* represent the expression values of two miRs), 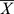 and 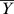 are their respective means and *S_x_* and *S_Y_* are the respective standard deviations, and *n* is the number of individuals or pairs of scores in the sample.

As we wanted to study the highly correlated miR pairs, we selected those pair of miRs which have correlation coefficient greater than a certain threshold (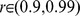). These pairs of miRs thus represent a highly correlated co-expression module which can have characteristic biological significance.

## Supporting Information

Figure S1 **TF-miR network for Group 1 miRs.** Figure shows the interaction between the highly significant 29 miRs from Group1and their respective TFs. Diamond nodes in the outer layer represent TFs and the circular nodes in the inner layer represent miRs. TF out-degree or miR in-degree can be visualized in this network where the direction of regulation is from TF to miR.
(TIF)

Figure S2 **TF-miR network for Group 2 miRs.** Figure shows the interaction between the highly significant 59 miRs from Group2 and their respective TFs. Diamond nodes in the outer layer represent TFs and the circular nodes in the inner layer represent miRs. TF out-degree or miR in-degree can be visualized in this network where the direction of regulation is from TF to miR.
(TIF)

Figure S3 **Multiple alignment of miR-200c across different species.** This information was obtained from miRNAviewer which presents a global view of homologous miR genes in many species [34]. Multiple alignment is colored gray for aligned sequences, red for mismatches and blue for mature miR region.
(TIF)

Figure S4 **Heatmap of the 204 DE miRs across 19 PD and 13 control samples.** Red blocks represent disease samples whereas green represents control samples. This figure was generated in MATLAB (R2012b).
(TIF)

Table S1 **Functional Enrichment analysis of the miR targets.** This file contains the information of top 20 over-representative GO Biological Processes associated with the mRNA targets of Group1 and Group2 miRs.
(DOCX)

Table S2 **Functional Enrichment analysis of the miR TFs.** This file contains the information of top 20 most significant KEGG Pathways associated with the TFs of Group1 and Group2 miRs.
(DOCX)

Table S3 **List of highly significant miRs from Group1 and Group2 which were used to create regulatory networks.** This file lists down 29 miRs from Group1 and 59 miRs from Group2 which were associated with the top 20 over-representative GO biological processes and later used to build up the respective regulatory networks.
(XLSX)

Table S4 **TF and mRNA target information for the 14 IR hub miRs.** TF information was obtained from TransmiR database and top 10 target information was obtained from TarMiR platform. The shared target list of three servers DIANA microT, miRanda and TargetScan were used to retrieve mRNA target information.
(XLSX)

Table S5 **Distribution of 204 DE miRs across 6 different hierarchical clusters.** This file contains the result of hierarchical clustering analysis in which cluster 2, 3, 4 and 6 appeared as the most significant miR clusters containing 62, 99, 25 and 14 miRs respectively.
(XLSX)

Table S6 **Top 20 most over-representative KEGG pathways associated with the four significant miR clusters obtained from hierarchical clustering analysis.**
(XLSX)

Table S7 **Result of the PhastCons analysis for the 14 co-expressed hub miRs which were not previously found to be linked with PD.** Conservation analysis performed with the PhastCons dataset in UCSC genome browser resulted in high PhastCons scores for most of the co-expressed hubs specially for hsa-miR-92a which was found to be common in both regulatory and co-expression networks.
(DOCX)

Table S8 **Clinical Information about the experimental group.** This file contains the clinical information of the 19 PD patients and 13 Control individuals as provided by the authors [73].
(XLSX)

Table S9 **List of 73 PD related miRs obtained through text mining.** 26 PD related miRs were obtained from HMDD and 47 more miRs were obtained from PubMed. In this way, 73 miRs were found to have association with PD. This file lists down the respective sources (either PMID or HMDD) of all these 73 PD related miRs.
(XLSX)
