# Supplementary figures and images for: Studying the System-Level Involvement of MicroRNAs in Parkinson's Disease
Source: PLoS One. 2014 Apr 1;9(4):e93751. doi: 10.1371/journal.pone.0093751 (PMC3972105; doi:10.1371/journal.pone.0093751)

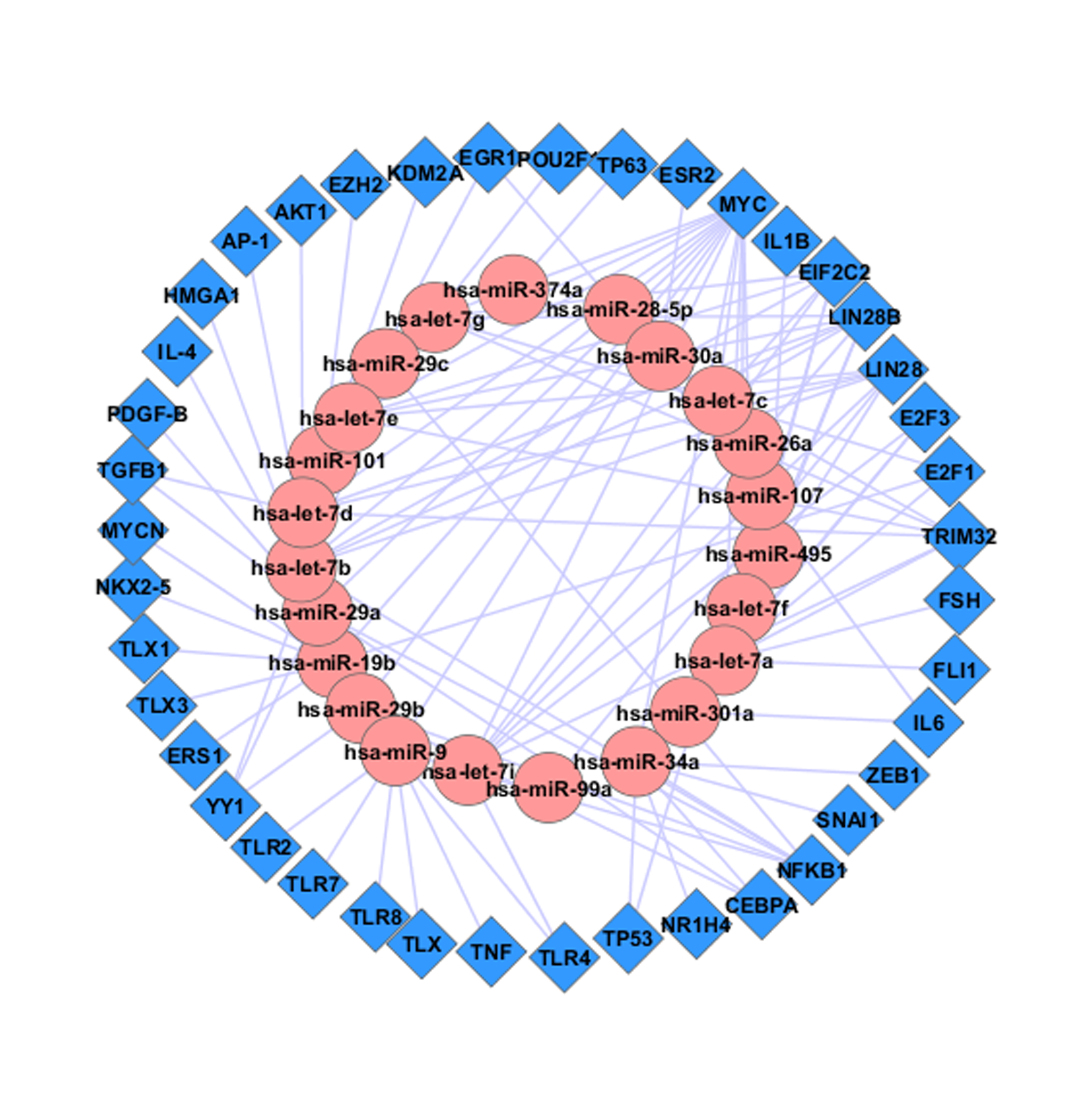

Supplement: Figure S1 — TF-miR network for Group 1 miRs. Figure shows the interaction between the highly significant 29 miRs from Group1and their respective TFs. Diamond nodes in the outer layer represent TFs and the circular nodes in the inner layer represent miRs. TF out-degree or miR in-degree can be visualized in this network where the direction of regulation is from TF to miR. (TIF) [file pone.0093751.s001.tif]

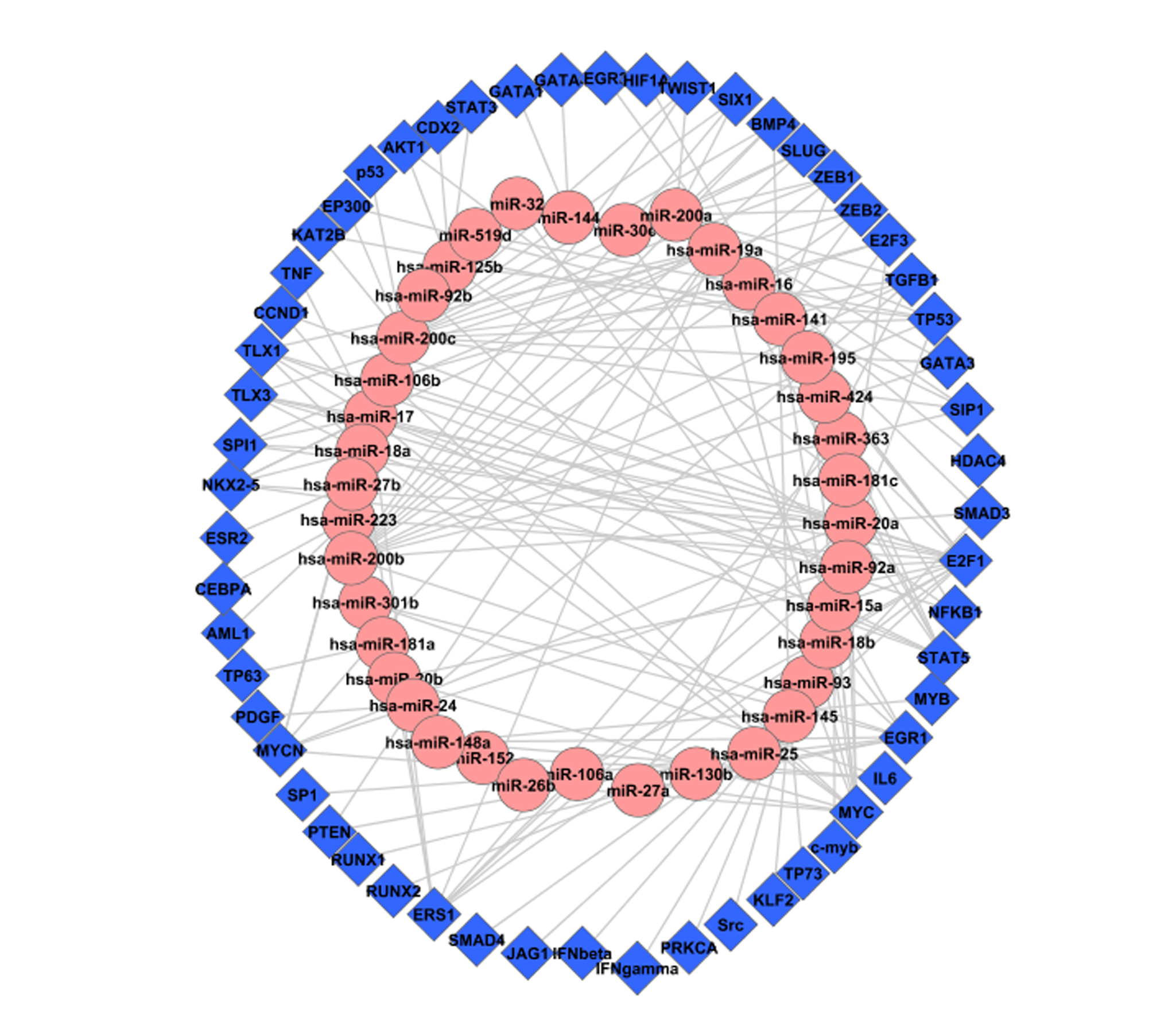

Supplement: Figure S2 — TF-miR network for Group 2 miRs. Figure shows the interaction between the highly significant 59 miRs from Group2 and their respective TFs. Diamond nodes in the outer layer represent TFs and the circular nodes in the inner layer represent miRs. TF out-degree or miR in-degree can be visualized in this network where the direction of regulation is from TF to miR. (TIF) [file pone.0093751.s002.tif]

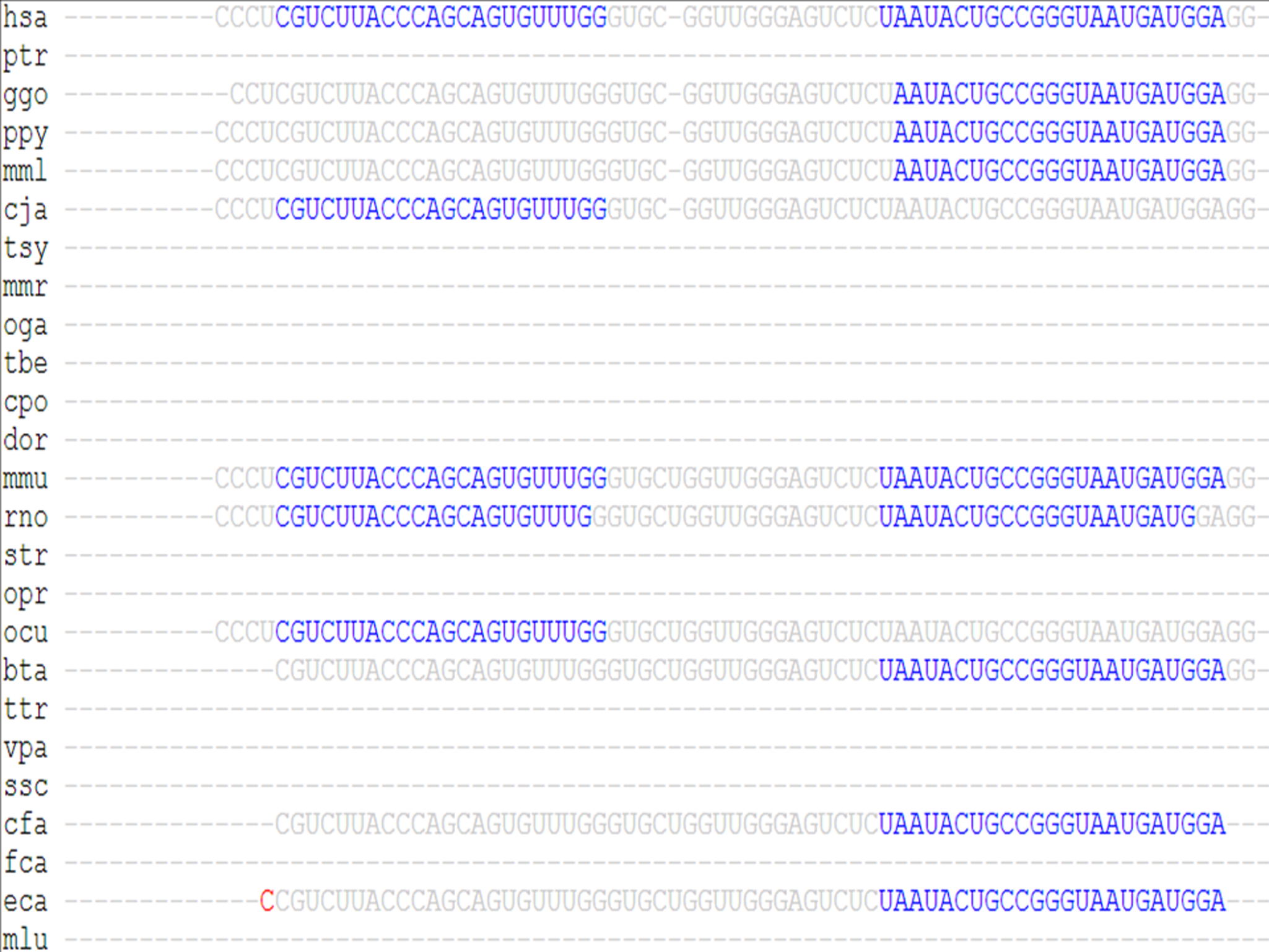

Supplement: Figure S3 — Multiple alignment of miR-200c across different species. This information was obtained from miRNAviewer which presents a global view of homologous miR genes in many species [34]. Multiple alignment is colored gray for aligned sequences, red for mismatches and blue for mature miR region. (TIF) [file pone.0093751.s003.tif]

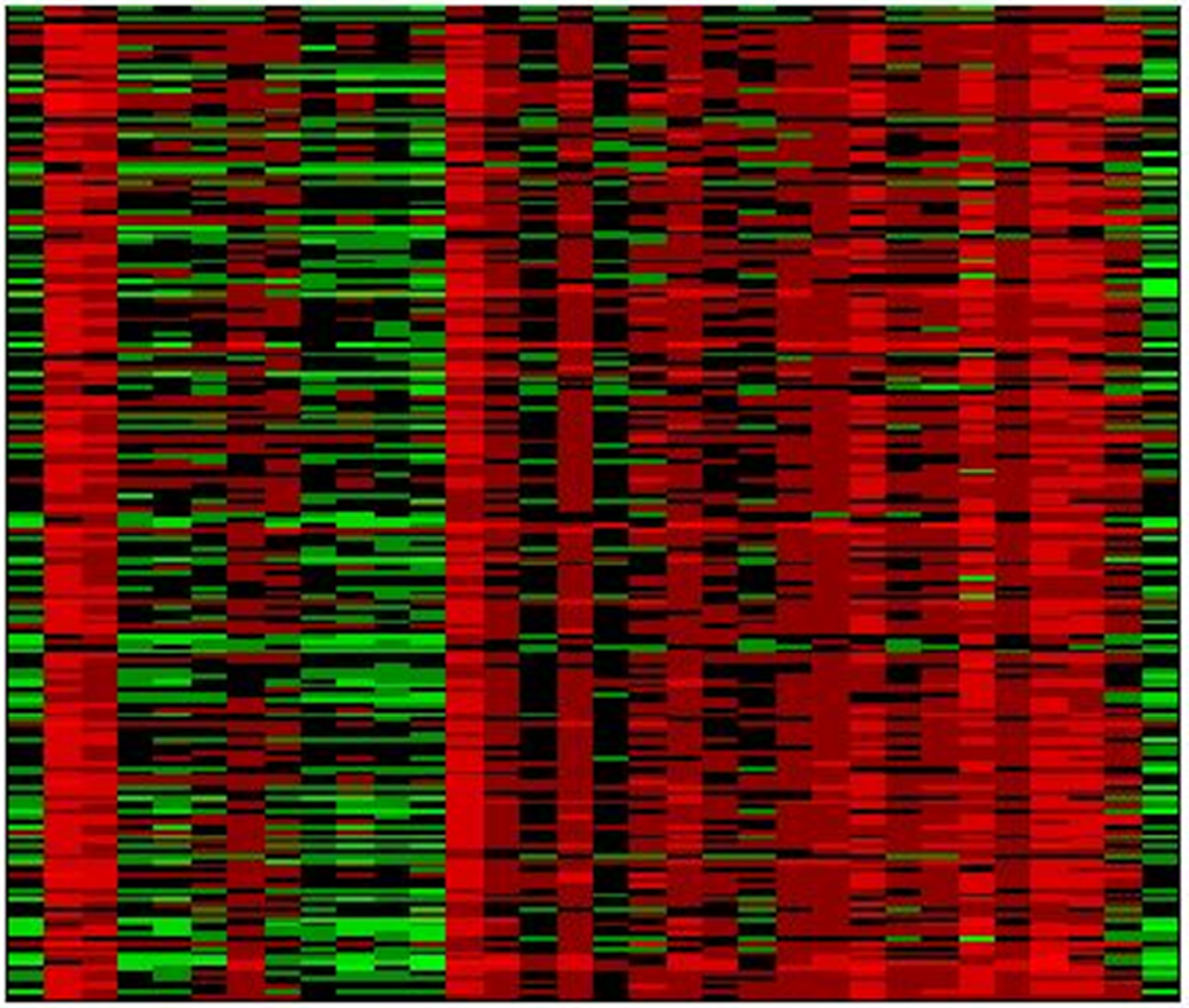

Supplement: Figure S4 — Heatmap of the 204 DE miRs across 19 PD and 13 control samples. Red blocks represent disease samples whereas green represents control samples. This figure was generated in MATLAB (R2012b). (TIF) [file pone.0093751.s004.tif]
