# Supplementary material for: Studying the System-Level Involvement of MicroRNAs in Parkinson's Disease
Source: PLoS One. 2014 Apr 1;9(4):e93751. doi: 10.1371/journal.pone.0093751 (PMC3972105; doi:10.1371/journal.pone.0093751)
Supplement: Table S1 — Functional Enrichment analysis of the miR targets. This file contains the information of top 20 over-representative GO Biological Processes associated with the mRNA targets of Group1 and Group2 miRs. (DOCX) [file pone.0093751.s005.docx]

**Table S1 : Top 20 most significant GO Biological Processes associated with the target mRNAs of Group1 and Group 2 miRs.**

| ID | Name | *p*-value | ID | Name | *p*-value |
| --- | --- | --- | --- | --- | --- |
| GO:0007167 | enzyme linked receptor protein signaling pathway | 7.85E-19 | GO:0007399 | regulation of neurogenesis | 6.54E-46 |
| GO:0048468 | cell development | 8.65E-19 | GO:0048468 | cell development | 6.56E-32 |
| GO:0010629 | negative regulation of gene expression | 1.13E-16 | GO:0030182 | neuron differentiation | 6.72E-29 |
| GO:0006366 | transcription from RNA polymerase II promoter | 1.16E-16 | GO:0006366 | transcription from RNA polymerase II promoter | 3.89E-28 |
| GO:0009887 | organ morphogenesis | 2.36E-16 | GO:0022008 | Neurogenesis | 4.43E-28 |
| GO:0006468 | protein phosphorylation | 1.64E-15 | GO:0048699 | generation of neurons | 2.24E-27 |
| GO:0016481 | negative regulation of transcription, DNA-dependent | 2.54E-15 | GO:0009893 | positive regulation of metabolism | 2.26E-27 |
| GO:0009890 | negative regulation of biosynthetic process | 3.15E-15 | GO:0006468 | protein phosphorylation | 1.66E-25 |
| GO:0009892 | negative regulation of metabolism | 1.92E-14 | GO:0010628 | positive regulation of gene expression | 5.12E-25 |
| GO:0000902 | cell morphogenesis | 2.69E-14 | GO:0010646 | regulation of cell communication | 1.22E-24 |
| GO:0009888 | tissue development | 2.94E-14 | GO:0007167 | enzyme linked receptor protein signaling pathway | 2.48E-24 |
| GO:0015031 | protein transport | 4.34E-14 | GO:0045941 | positive regulation of transcription | 3.73E-24 |
| GO:0051270 | regulation of cellular component movement | 5.06E-14 | GO:0009887 | organ morphogenesis | 6.56E-24 |
| GO:0030182 | neuron differentiation | 7.46E-14 | GO:0048666 | neuron development | 1.69E-23 |
| GO:0006928 | cellular component movement | 1.41E-13 | GO:0031328 | positive regulation of cellular biosynthetic process | 2.55E-23 |
| GO:0009893 | positive regulation of metabolic process | 3.08E-13 | GO:0010629 | negative regulation of gene expression | 4.88E-23 |
| GO:0022008 | neurogenesis | 3.29E-13 | GO:0030030 | cell projection organization | 2.87E-22 |
| GO:0016477 | cell migration | 4.08E-13 | GO:0000904 | cell morphogenesis involved in differentiation | 3.71E-22 |
| GO:0030334 | regulation of cell migration | 6.83E-13 | GO:0016481 | negative regulation of transcription, DNA-dependent | 6.47E-22 |
| GO:0051246 | regulation of protein metabolism | 8.68E-13 | GO:0009890 | negative regulation of biosynthetic process | 1.34E-21 |
