# Supplementary material for: Studying the System-Level Involvement of MicroRNAs in Parkinson's Disease
Source: PLoS One. 2014 Apr 1;9(4):e93751. doi: 10.1371/journal.pone.0093751 (PMC3972105; doi:10.1371/journal.pone.0093751)
Supplement: Table S2 — Functional Enrichment analysis of the miR TFs. This file contains the information of top 20 most significant KEGG Pathways associated with the TFs of Group1 and Group2 miRs. (DOCX) [file pone.0093751.s006.docx]

**Table S2 : Functional Enrichment analysis of the miR TFs - top 20 most significant KEGG Pathways associated with the TFs of Group1 and Group2 miRs**

| Group1 | | | Group2 | | |
| --- | --- | --- | --- | --- | --- |
| ID | **Name** | ***p*-value** | **ID** | **Name** | ***p*-value** |
| hsa04620 | Toll-like receptor signaling pathway | 2.10E-19 | **hsa05200** | Pathways in cancer | 2.41E-29 |
| hsa05222 | Small cell lung cancer | 2.75E-16 | **hsa05220** | Chronic myelogenous leukemia (CML) | 4.32E-24 |
| hsa05220 | Chronic myelogenous leukemia (CML) | 1.02E-14 | **hsa05222** | Small cell lung cancer | 1.44E-22 |
| hsa05212 | Pancreatic cancer | 3.72E-13 | **hsa05212** | Pancreatic cancer | 6.03E-22 |
| hsa05219 | bladder cancer | 1.35E-12 | **hsa04110** | Cell cycle | 6.18E-21 |
| hsa04210 | Apoptosis | 2.14E-12 | **hsa05215** | Prostate cancer | 8.34E-19 |
| hsa05215 | Prostate cancer | 2.67E-12 | **hsa05214** | Glioma | 4.38E-18 |
| hsa05200 | Pathways in cancer | 3.72E-12 | **hsa04310** | Wnt signaling pathway | 1.29E-17 |
| hsa05223 | Non-small cell lung cancer, | 1.73E-11 | **hsa05221** | Acute myeloid leukemia (AML) | 5.26E-17 |
| hsa04621 | NOD-like receptor signaling pathway | 1.73E-11 | **hsa05223** | Non-small cell lung cancer, | 3.04E-16 |
| hsa04110 | Cell cycle | 2.35E-11 | **hsa05219** | bladder cancer | 6.87E-16 |
| hsa04010 | MAPK signaling pathway | 2.99E-11 | **hsa05218** | Melanoma | 1.13E-15 |
| hsa05214 | Glioma | 3.27E-11 | **hsa04630** | Jak-STAT signaling pathway | 1.51E-15 |
| hsa05218 | Melanoma | 4.16E-11 | **hsa05210** | colorectal cancer | 3.58E-15 |
| hsa05020 | Prion diseases | 2.22E-10 | **hsa04350** | TGF-beta signaling pathway | 4.18E-15 |
| hsa05213 | Endometrial cancer | 4.60E-10 | **hsa05213** | Endometrial cancer | 4.81E-15 |
| hsa05221 | Acute myeloid leukemia (AML) | 5.26E-10 | **hsa04530** | Tight junction | 3.98E-13 |
| hsa04660 | T cell receptor signaling pathway | 7.64E-10 | **hsa04510** | Focal adhesion | 1.34E-12 |
| hsa04623 | Cytosolic DNA-sensing pathway | 1.25E-09 | **hsa05216** | Thyroid cancer | 2.44E-12 |
| hsa05216 | Thyroid cancer | 3.96E-09 | **hsa04115** | p53 signaling pathway | 3.60E-12 |
