# Supplementary material for: Studying the System-Level Involvement of MicroRNAs in Parkinson's Disease
Source: PLoS One. 2014 Apr 1;9(4):e93751. doi: 10.1371/journal.pone.0093751 (PMC3972105; doi:10.1371/journal.pone.0093751)
Supplement: Table S7 — Result of the PhastCons analysis for the 14 co-expressed hub miRs which were not previously found to be linked with PD. Conservation analysis performed with the PhastCons dataset in UCSC genome browser resulted in high PhastCons scores for most of the co-expressed hubs specially for hsa-miR-92a which was found to be common in both regulatory and co-expression networks. (DOCX) [file pone.0093751.s011.docx]

**Table S7 : Summary statistics for the conservation analysis obtained from the PhastCons dataset of UCSC Genome Browser** (*http://genome.ucsc.edu/*) **for the 14 co-expressed hub miRs (which are not previously linked with PD)** [32].

| miR name | Position (obtained from miRBase) [35] | phastCons Score | | |
| --- | --- | --- | --- | --- |
|  |  | **Smallest** | **Biggest** | **Average** |
| hsa-miR-190 | chr15: 63116156-63116240 | 183 | 1000 | 332 |
| hsa-miR-155 | chr21: 26946292-26946356 | 602 | 602 | 602 |
| hsa-miR-148a | chr7: 25989539-25989606 | 582 | 582 | 582 |
| hsa-miR-92a* | chr13: 92003568-92003645 | 750 | 750 | 750 |
| hsa-miR-338-3p | chr17: 79099683-79099749 | 472 | 497 | 485 |
| hsa-miR-143 | chr5: 148808481-148808586 | 588 | 588 | 588 |
| hsa-miR-181a-2 | chr9: 127454721-127454830 | 599 | 599 | 599 |
| hsa-miR-30d | chr8: 135817119-135817188 | 489 | 511 | 500 |
| hsa-miR-589 | chr7: 5535450-5535548 | 248 | 248 | 248 |
| hsa-miR-148b | chr12: 54731000-54731098 | 319 | 638 | 479 |
| hsa-miR-15a | chr13: 50623255-50623337 | 624 | 624 | 624 |
| hsa-miR-192 | chr11: 64658609-64658718 | 488 | 503 | 496 |
| hsa-miR-27b | chr9: 97847727-97847823 | 643 | 643 | 643 |
| hsa-miR-548c-5p | chr12: 65016289-65016385 | -- | -- | -- |

* **hsa-miR -92a appeared as a common hub in both regulatory and co-expression network.**
